# Beyond Short Microhomologies: Mismatch‐Compatible Pol θ‐Mediated DNA Damage Repair

**DOI:** 10.1002/bies.70129

**Published:** 2026-03-25

**Authors:** Yuzhen Li, Richard D. Wood

**Affiliations:** ^1^ Department of Epigenetics and Molecular Carcinogenesis The University of Texas MD Anderson Cancer Center Houston Texas USA

**Keywords:** DNA polymerase θ (Pol θ), microhomology, mismatches, mutational signatures, TMEJ

## Abstract

DNA polymerase θ (Pol θ)‐mediated end‐joining (TMEJ), one of several pathways for repairing DNA double‐strand breaks, is traditionally thought to initiate via anchoring at short, consecutive, and perfectly matched microhomologies (MHs). Emerging evidence indicates that Pol θ can utilize MHs containing mismatches both in vitro and in vivo. This revised definition of MH provides a mechanistic explanation for a broader spectrum of Pol θ‐dependent repair outcomes. Here, we summarize recent findings on the revised definition of MHs utilized by Pol θ, assess the applicability of this concept across species, and compare TMEJ with other (micro)hom(e)ology‐mediated repair pathways. We explore how mismatch‐containing MHs expand Pol θ‐associated mutational signatures and provide a framework for future studies on Pol θ’s role in DNA repair and cancer biology.

## Graphical abstract

Pol θ is traditionally thought to use a short, perfectly matched microhomology (MH) to initiate end‐joining repair of a DNA double‐strand break. Emerging evidence indicates that Pol θ generally utilizes mismatch‐compatible longer microhomologies. This revised definition of MH is applicable across species and broadens the definition of Pol θ‐associated mutational signatures.

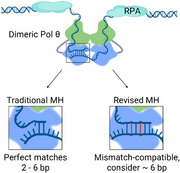

## 1 Introduction

**FIGURE 1:**
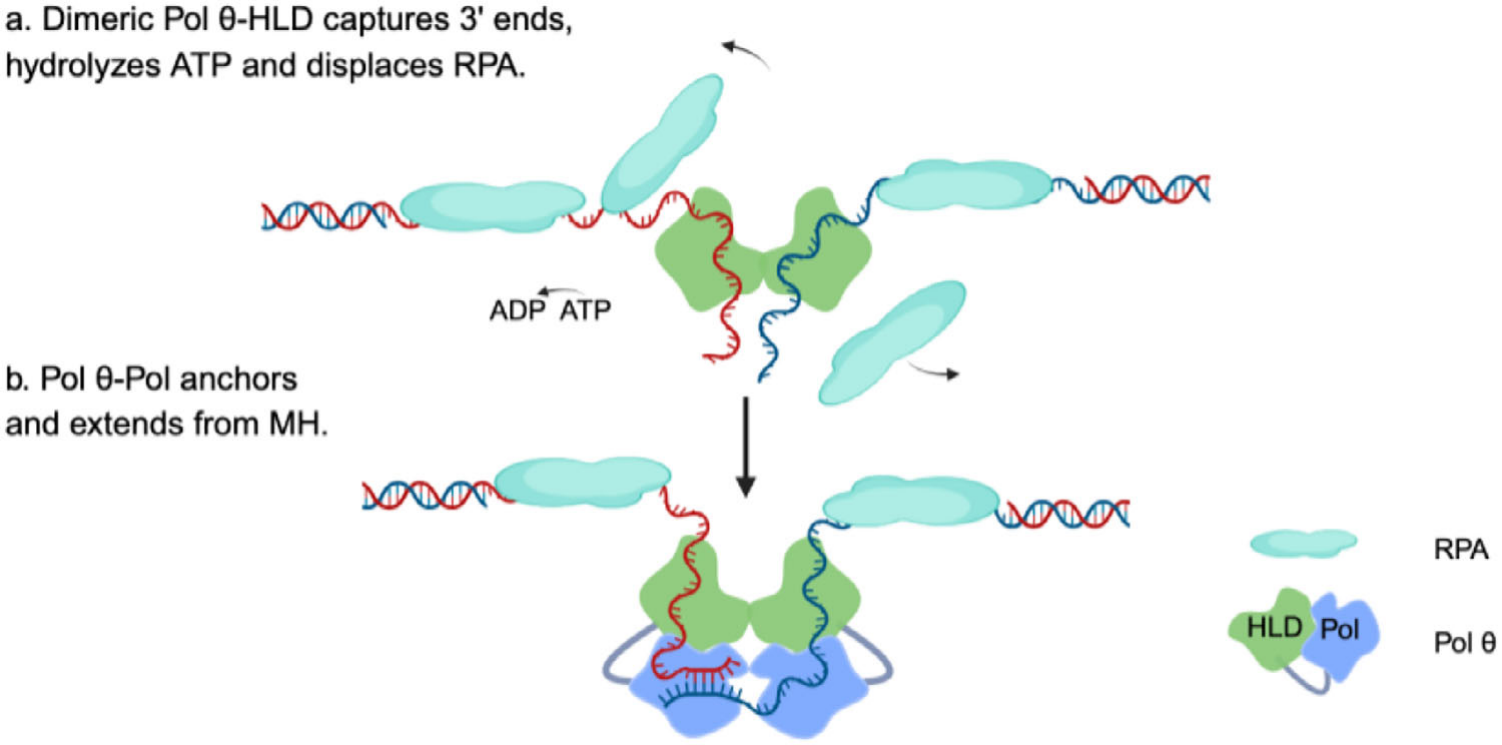
Working model of Pol θ helicase‐like domain (HLD) and polymerase domain (Pol) during theta‐mediated end‐joining. (a) During TMEJ, Pol θ HLD forms a dimer with each HLD monomer engaging a ssDNA tail generated at a DSB site. This HLD dimeric positions one 3′ end in proximity to one another. If the two emerging 3′ ends have sufficient base pairing, they can potentially pair (anneal). In most cases, under physiological cellular conditions, the available MH is not sufficient to form a stable duplex by base‐pairing. (b) A subsequent step is necessary so that the MH can be selected and extended by the DNA polymerase domain of Pol θ, which uses one strand as primer. The exact path of ssDNA through the Pol domain of Pol θ is not yet known. Here we illustrate a path where one 3′ tail passes through one Pol domain to meet the other tail in a second Pol domain to allow MH searching and anchoring. This is inspired by the dimeric crystal structure of bacterium G2L4 reverse transcriptase that extends from microhomologies [13].

DNA polymerase theta (Pol θ, gene name *POLQ*), the central effector of theta‐mediated end joining (TMEJ) was first reported to function in double‐strand break (DSB) repair in 2010 [1], following the discovery in 1990 of its Drosophila ortholog, mus308, which functions in DNA cross‐link repair [2]. During TMEJ, Pol θ functions as a dimer [3] that displaces replication protein A (RPA) from single‐stranded DNAs (ssDNAs) and independently captures two ssDNA tails via its dimeric N‐terminal helicase‐like domain, positioning the 3′ end in proximity to one another [4, 5]. This process is termed “strand‐capture” [6, 7] (Figure 1a). If sufficient base pairing exists between the two 3′ ends, potential pairing (annealing) may occur, although this is rare under physiological conditions. Subsequently, the two ssDNAs meet in the polymerase domain (Pol) of Pol θ where the microhomology (MH) search finds an anchoring position, defined as a MH pairing position that allows extension from a 3′ end [8] (Figure 1b). If neither ssDNA tail can server as a productive primer, the dissociated 3′ tail can enter the exonuclease site of Pol δ to be trimmed, followed by re‐engagement with the Pol domain of Pol θ for another round of MH searching and anchoring [9, 10].

Microhomology searching and anchoring by Pol θ is a critical step for the end‐joining process. Traditionally, microhomologies used during TMEJ are defined as short (2 – 6 bp), perfectly matched sequences between two ssDNA tails at DSB sites [11, 12]. However, emerging evidence indicates that Pol θ can utilize imperfect or mismatched microhomologies during TMEJ [8]. Despite its central role in TMEJ, more understanding is needed of factors that contribute to MH selection and anchoring by Pol θ. Characterizing these determinants could advance our understanding of TMEJ mechanism and its biological consequences.

The concept of microhomology predates its connection to Pol θ. It was first reported in studies of end‐joining between two 3′ tails of transfected linear plasmids in monkey cell lines in 1986 [14]. Following decades of research, multiple repair pathways were found to utilize (micro)homology, including microhomology‐mediated break‐induced replication (MMBIR), a one‐ended DSB repair pathway that uses a microhomologous sequence as a template for repair [15, 16]. MMBIR has been proposed to cause copy number variation and, ultimately, to contribute to the formation or expansion of low copy repeats in genomic disorders [15, 17]. Notably, MMBIR exists in both Pol θ containing species [18, 19] and species lacking Pol θ, such as yeast [15, 16, 20]. Moreover, the concept of homeology (paired sequences containing mismatches or gaps) has also emerged from studying the sequence context of genome rearrangements and repair junctions [17, 18, 21]. Repair junctions likely derived from micro stretches of homeologies are enriched in clinical samples [18, 22]. Indeed, Pol θ‐mediated repair can preserve genome integrity by preventing large‐scale genomic damage [11, 23]. Nevertheless, *POLQ* is one of a set of genes whose high expression is associated with poor prognosis in multiple cancer types [24, 25, 26, 27, 28].

This review explores recent advances in the understanding of MH anchoring during TMEJ and proposes a revised definition of MH to include mismatches. We first address characteristics of microhomologies including mismatch tolerance, length of the MH, and how the two ends are handled during TMEJ. Then we highlight mechanistic differences between TMEJ and other “(micro)homology” mediated repair pathways such as MMBIR. Finally, we discuss the implications of these findings for DNA damage repair, genome stability, and potential cancer therapies.

## 2 Microhomology with Mismatches in TMEJ: A Revised Definition

**FIGURE 2:**
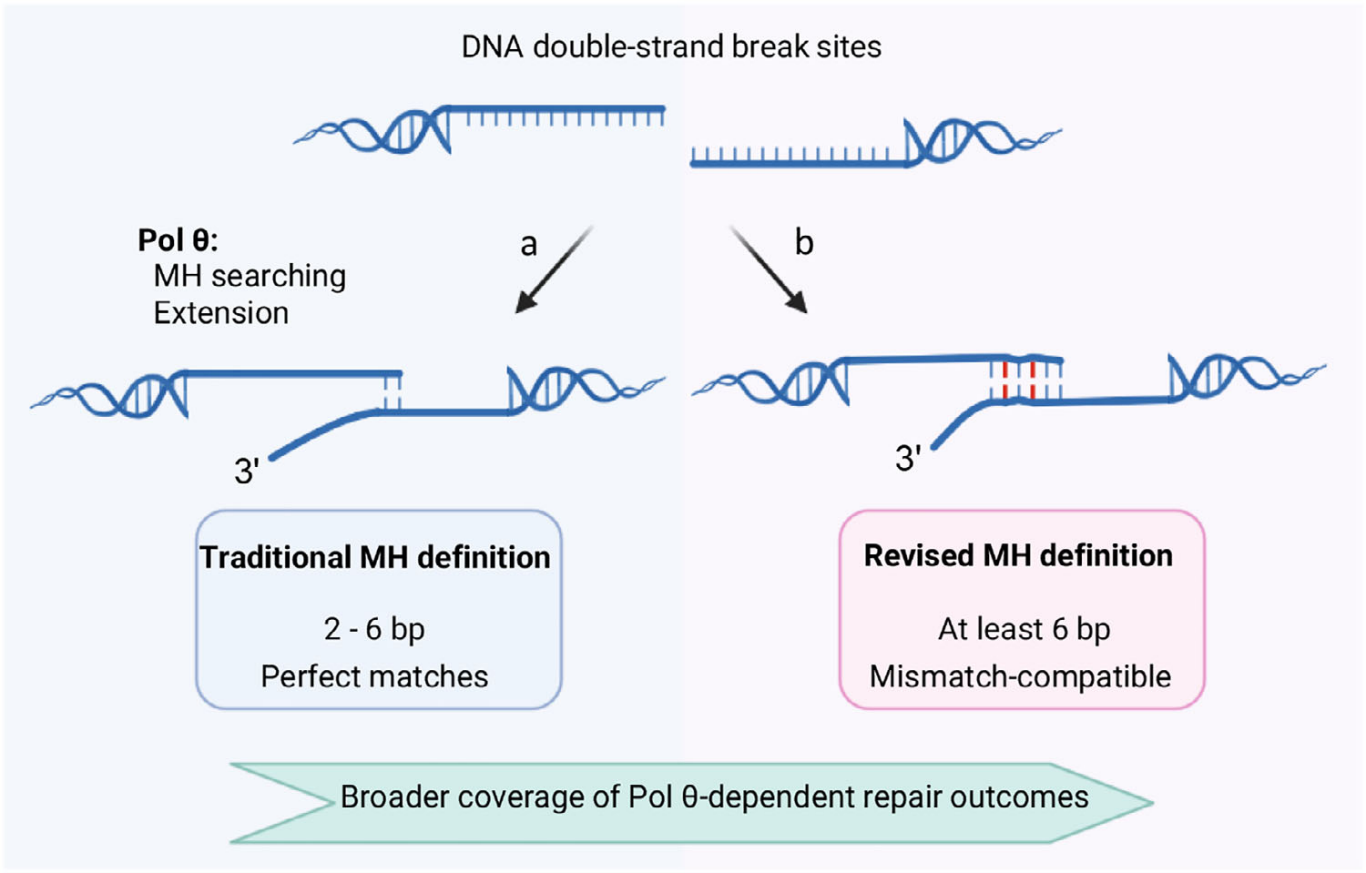
Comparison of traditional and revised MH definition during TMEJ. (a) Traditionally defined MHs anchored by Pol θ are generally internal, short and composed of consecutive paired bases between two ssDNA tails at DSB sites. (b) A revised definition of MH includes mismatches, sometimes longer. At least 6 bp near the 3′ terminus contributes to functional MH anchoring but can accommodate mismatches (shown in pink box).

Microhomology has traditionally been defined as short, consecutive base pairing at DNA repair junctions. Short paired bases within 15 nt between two tails of a linear plasmid were found in 97% of analyzed junctions without insertions in end‐joining studies in mammalian cells [14]. Subsequent analyses across diverse organisms‐including *Drosophila melanogaster* [1, 29], *Arabidopsis thaliana* [30, 31], *Homo sapiens* [32, 33, 34], and *Caenorhabditis elegans* [35, 36, 37] reinforced this framework by identifying internal, short, and apparently consecutive paired bases of variable length at repair junctions associated with Pol θ‐mediated end joining (Figure 2a).

Several Pol θ‐dependent repair outcomes are difficult to reconcile with a strict requirement for short, perfectly matched microhomologies. In *Drosophila*, Pol θ can utilize longer microhomologies during repair of a site‐specific DSB formed via P transposase [1, 29]. The favored 8 bp microhomology contains a single internal mismatch. In some mammalian cell lines, CRISPR/Cas9‐induced DSBs repaired by Pol θ sometimes yield junctions interpreted as having 1 bp MH or no detectable MH [34, 38]. In vitro studies further support this flexibility. The purified human Pol θ polymerase domain can mediate end‐joining when a designed microhomology between two 3′ tails is positioned 3 nucleotides away from the 3′ terminus [39]. Moreover, Pol θ can initiate DNA synthesis using only a few paired bases [40]. Together, these observations suggest that Pol θ does not strictly require perfectly matched microhomologies to initiate repair (Figure 2b).

In line with this, our recent work used high‐throughput sequencing combined with bioinformatic analysis to demonstrate that Pol θ frequently utilizes microhomologies containing mismatches rather than perfectly paired sequences [8]. Mechanistically, mismatch tolerance during TMEJ is partially facilitated by specific primer‐grasp amino acids within the polymerase domain of Pol θ [9]. For MH anchoring, pairing at the 3′ terminus of the priming strand is critical, while at least two matches within the remaining five nucleotides also increase the probability of successful MH anchoring [8]. Importantly, microhomology anchoring by Pol θ appears to be governed by the density of matched pairs rather than the matches being consecutive.

Together, these findings support a revised definition of microhomology in TMEJ. Rather than short, perfectly matched, and consecutive sequences, Pol θ recognizes and anchors imperfect microhomologies composed of non‐consecutive matches that satisfy minimal primer‐template pairing requirements. This expanded definition provides a mechanistic explanation for in vivo repair outcomes lacking traditionally defined microhomology and highlights the remarkable substrate flexibility of Pol θ during end‐joining [8, 34, 38]. Preferential utilization of mismatch‐compatible microhomologies by Pol θ may promote timely end‐joining once resection has occurred, rather than prolonged searching for fully matched MHs. Early anchoring at suboptimal but more proximal MHs could limit the need of additional processing steps like Pol δ trimming and reduce extensive deletions that may be particularly deleterious to genome stability.

## 3 MH Length in TMEJ: Beyond Short Consecutive Sequences

Pol θ is widely conserved and expressed across metazoans and plants [11, 41], yet the apparent length and context of microhomologies at Pol θ‐dependent repair junctions vary substantially among organisms. In *C. elegans*, Pol θ has been implicated in deletions induced by replication‐blocking G‐quadruplexes or psoralen‐interstrand crosslinks, which frequently display minimal 1 bp microhomology at junctions [35, 36]. Subsequent studies showed that deletions containing 1 – 4 bp of microhomology at junctions are dependent on Pol θ activity in *C. elegans* [37]. These junctions may arise from theta‐mediated end‐joining acting on double‐strand breaks generated either after replication fork collapsed or following the conversion of post‐replication ssDNA gaps into DSBs, as illustrated in Figure 4. In contrast, studies in *Drosophila* reveal a prominent contribution of longer microhomologies. In a *spn‐A (Rad51)* mutant background, Pol θ‐dependent DSB repair is frequent. Loss of *PolQ* in this background significantly reduces junctions involving annealing at 5 – 10 bp MHs [1]. Notably, transgenic flies expressing an ATPase‐dead Pol θ variant retained high usage of an imperfect internal 8‐bp MH but showed reduced repair events mediated by >3 bp MHs [29]. Mammalian and plant systems further support a broader view of microhomology length. In mouse embryonic fibroblasts, Pol θ‐enriched deletions were associated with 2 – 6 bp microhomologies located within 10 – 15 bp of the break site [33]. In *Arabidopsis*, Pol θ deficiency reduced T‐DNA capture events characterized by junctional microhomologies with median lengths of 3 bp and 1 bp on the left and right borders, respectively [31]. While these could constitute short MH under traditional criteria, they may obscure the true extent of pairing information used by Pol θ during TMEJ.

**FIGURE 3:**
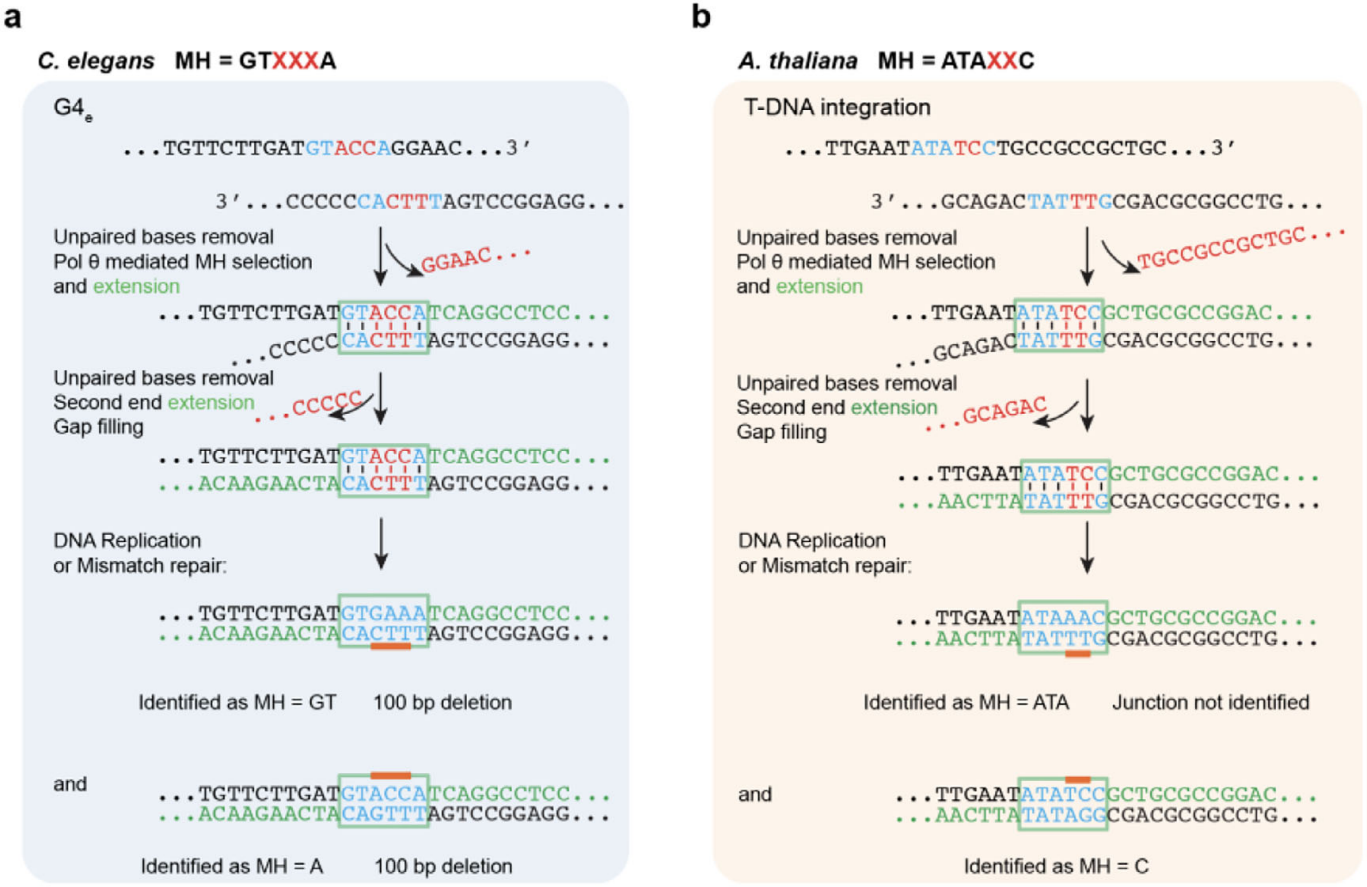
Examples of how mismatched MHs can redefine MH pairing sites for Pol θ‐mediated repair in *C. elegans* (a) and *A. thaliana* (b). At the top of each panel, partial sequences of two 3ʹ single‐stranded DNAs involved in Pol θ‐mediated end‐joining are shown. Matched bases in the MH are colored blue, and mismatched bases are colored red. Flanking unpaired bases are removed by nuclease action [10]. Partially extended sequences are indicated by bases colored green. The internally mismatched bases may be resolved by downstream DNA replication or mismatch repair, yielding the two different potential outcomes shown at the bottom. (a) G4_e_‐derived mutagenesis depending on Pol θ‐mediated repair in *C. elegans* from [35]. The G4_e_ sequence (G_3_AG_5_TG_5_AG_8_) is predicted to form a G4 structure. Two deletion events potentially originated from the same internally mismatched MH with 3 paired bases. These events were previously scored as two different 100 bp deletions with MH lengths of 1 or 2. (b) *Agrobacterium* T‐DNA integration into *A. thaliana* [30]. Similar internally mismatched MH with 4 paired bases can produce two potential different outcomes, one known to depend on Pol θ.

Under a revised definition of microhomology, non‐consecutive matches, and mismatches are permitted (Figure 1b). Accordingly, many Pol θ‐dependent repair outcomes previously classified as having minimal or no microhomology can be reinterpreted as involving longer mismatch‐containing MH. In human K562 cells, major deletion outcomes lacking obvious microhomology by traditional definitions [34] can be interpreted by longer internal mismatch‐containing MH [8]. This is also true for some deletions observed in mouse embryonic stem cells [38]. Moreover, two distinct Pol θ‐dependent deletion outcomes of CRISPR/Cas9‐introduced DSBs can sometimes be traced back to anchoring at a shared mismatch‐containing microhomology, followed by divergence through different downstream processing steps [8, 34]. In this way, a single anchoring event may increase deletion complexity arising from a break site. For example, comparable microhomology features can be observed by reanalysis of specific Pol θ‐dependent events in *A. thaliana* and *C. elegans* (Figure 3).

This ability of Pol θ to select mismatched microhomologies is supported by biochemical and structural properties of the polymerase. Purified human full‐length Pol θ can extend primers bearing mismatched primer‐template base pairs [40, 42]. Structural analyses of Pol θ polymerase domain shows extensive contacts with the phosphodiester backbone spanning the last six nucleotides of the primer [23, 43]. The compatibility of mismatches within MH by Pol θ is related to Pol θ specific primer‐grasp amino acids [9]. These primer‐grasp residues are well conserved in *POLQ* across species. For example, zebrafish Pol θ shows TMEJ and trans‐lesion bypass activities similar to those of human Pol θ [44].

Taken together, these findings suggest that there is more flexibility in microhomology selection than previously appreciated. MHs engaged during TMEJ are often longer and more frequently contain mismatches, differing from the short, perfectly matched sequences previously inferred. This mechanistically expanded view of microhomology provides a framework for expanded detection of Pol θ‐mediated events. This will help in understanding how Pol θ‐mediated repair promotes mutational diversity, contributes to genome instability, and may contribute to tumorigenesis.

## 4 Pol θ functions in TMEJ and can act on other substrates

Pol θ has long been recognized as a central component of TMEJ, a DSB repair pathway traditionally thought to involve two DNA tails. TMEJ can join two single‐stranded DNA tails generated by end resection (spanning ∼ 40 – 70 bases). This repair mode involving two DNA tails has been demonstrated using partially duplexed DNA substrates transfected into mammalian cells [10, 32, 33, 45], analyses of repair of DSBs generated by CRISPR/Cas9 or restriction endonucleases in mammalian cells [10, 38, 45, 46], in vitro reconstitution with purified Pol θ and designed DNA substrate mimics [8, 9, 47] and cytological detection of joining between two tails during mitosis [48, 49, 50]. However, Pol θ can synthesize DNA in reactions that do not involve two single strand tails. For example, Pol θ can perform primer extension, trans‐lesion synthesis, stem loop extension on ssDNA, and fill gaps in circular ssDNA [9, 47, 63].

**FIGURE 4:**
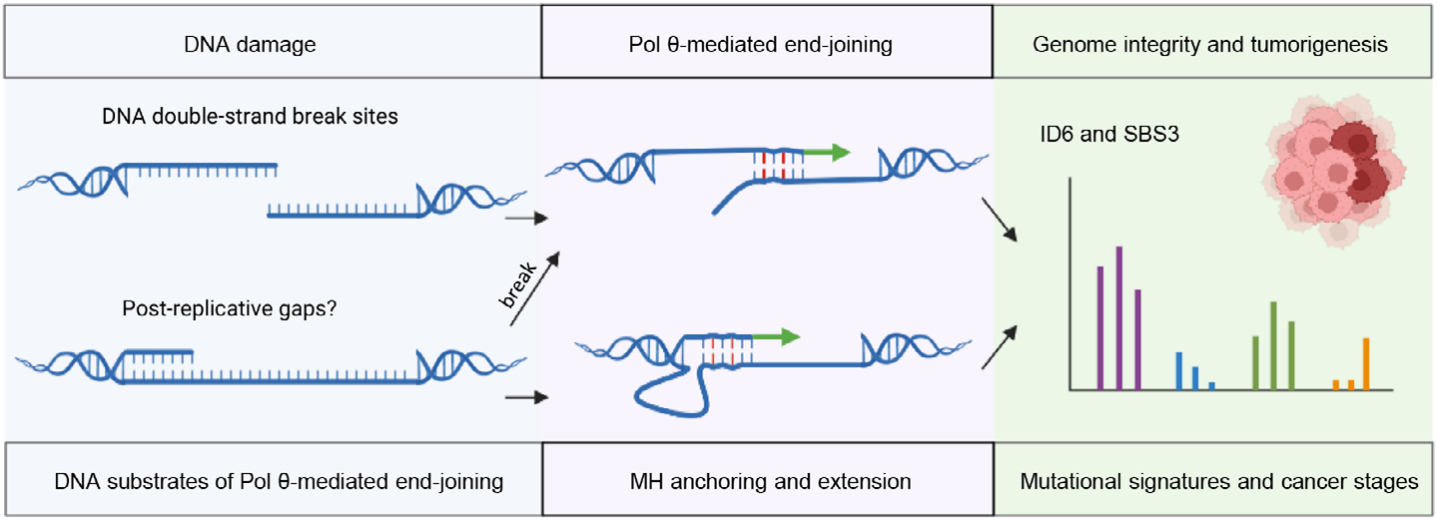
DNA substrates, MH anchoring and the influence of Pol θ‐mediated end‐joining on cell genome and fate. Pol θ can utilize mismatch compatible MHs between two ssDNAs at DSBs, or between a stalled primer and a post‐replicative ssDNA gap. Resolution of these mismatches can generate or modify cancer associated mutational signatures such as the small insertion and deletion signature 6 (ID6) and single base substitution signature 3 (SBS3), which correlate with Pol θ expression in HR‐defective cells.

Beyond its role in TMEJ, Pol θ has been suggested to function in the post‐replicative filling of ssDNA gaps in homologous recombination‐deficient cells under replication stress (Figure 4) [51, 52, 53]. One proposed mechanism is microhomology‐mediated gap skipping, in which Pol θ utilizes internal MHs to seal gaps through MHs formed between a ssDNA tail and sequence within the ssDNA gap [51]. An alternative interpretation for involvement of Pol θ could be that post‐replicative ssDNA gaps are cut by a nuclease, converting the gapped structure to a two‐ended DSB that can be repaired by TMEJ. Indeed, Mann et al. [53] showed that accumulation of DSBs in BRCA‐deficient cells is significantly increased upon Pol θ inhibition, but this increase is suppressed by loss of Mre11/NBS1/CtIP nuclease. Consistent with this view, Pol θ has been reported to repair clustered interstrand crosslinks (ICLs) by end‐joining two DNA tails generated during classical ICL processing [54]. Hence, the endogenous substrates processed by Pol θ in the post‐replicative filling of ssDNA gaps remain to be fully defined.

## 5 Other (Micro)hom(e)ology‐Mediated Repair Pathways

Numerous microhomology mediated end‐joining (MMEJ)‐like events have been reported in different species including those lacking Pol θ. For example, budding yeast does not possess Pol θ, nevertheless, MMEJ is observed and mediated by the polymerases Pol δ and Pol λ [55, 56]. A process of interchromosomal template switching can occur in which the partially copied DNA strand dissociates and pairs with a new template via a short stretch of perfectly matching bases (microhomology), resuming copying. Dalin et al. [57] showed that such apparent microhomology‐mediated template switching events involve imperfectly matched regions of ∼200 bp. Alternative end‐joining (A‐EJ) has also been identified in *E. coli*, where microhomology usage is prevalent despite the absence of Pol θ [58, 59].

**FIGURE 5:**
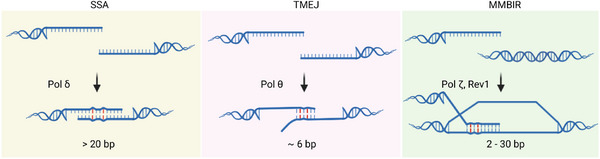
(Micro)hom(e)ology‐mediated repair pathways. All these pathways involve mismatch‐compatible (micro)homologies and engage different DNA polymerases. The homology lengths differ. The tolerance to mismatches within (micro)homologous sequences requires further investigation.

Even in species with Pol θ, microhomology‐mediated repair events can proceed via different mechanisms. Single‐strand annealing (SSA) is a subtype of homologous recombination (HR) that generates deletions without strand invasion. In SSA, microhomologies exceeding 20 bp are usually annealed and regulated in a RAD52 dependent process (Figure 5) [11]. MMBIR can initiate repair at microhomologies, typically defined as 2–30 bp, producing long tracts of synthesis via strand invasion at a large gap to rescue a stalled or collapsed replication fork (Figure 5) [16]. MMBIR is RAD51‐independent whereas canonical break‐induced replication (BIR) requires longer homology and RAD51 [17]. In yeast, MMBIR appears to be mediated by Pol ζ and Rev1 via 0 – 6 nt homology [60].

The presence of mismatches in TMEJ is mirrored and finds precedence in other (micro)homology‐mediated end‐joining pathways, suggesting a common flexibility in substrate usage. Analyses of MMBIR‐initiating templated insertions in human genomes have used the concept of microhomeology, defined as microhomology containing a single mismatch or gap. In lung adenocarcinoma, such microhomeologies account for more junctions than perfectly matched MHs [18]. In yeast, homeology severely reduced repair efficiency by inhibiting strand invasion and BIR [61]. In human clinical samples, breakpoint junctions of multiple de novo copy number variants produced by replication‐based mechanism such as MMBIR are enriched for microhomeologous sequences [22]. MMBIR involves extensive DNA replication via microhomology or microhomeology resulting in structural variants formation and regional hypermutation (Figure 5) [19].

In *Drosophila*, Pol θ does not act directly in the major homologous recombination pathway of synthesis‐dependent strand annealing (SDSA). Instead, Pol θ works in end‐joining repair following aborted SDSA [1]. Similarly, in *C. elegans*, Pol θ‐mediated end‐joining acts on failed SDSA intermediates [62]. However, Pol θ might function in other repair processes. Pol θ can fill gaps in vitro [63] and perhaps in vivo as discussed above, and can extend from microhomologies containing mismatches. MMBIR proceeds with multiple template switches at microhomologies or microhomeologies [60]. Further investigation could be done to determine whether Pol θ is involved in MMBIR in mammalian cells.

Besides MMBIR, homeology is also identified in other homology‐driven processes. Meiotic recombination is initiated between strands from two different parents. While some mismatched recombination events are rejected, many DMC1‐dependent events tolerate mismatched homologies [21, 64]. Further, nonallelic homologous recombination (NAHR), arising from nonallelic pairing of paralogous sequences such as retroelements can lead to deletions, duplications and inversions [17].

The boundaries between all these pathways are not always clear‐cut, suggesting that cells may switch among them according to the specific cellular and genomic context.

## 6 Summary and future directions

Pol θ‐mediated end‐joining initially depends on MH anchoring based on pairing information between two ssDNA tails. Recent studies have shown that MHs containing mismatches can account for more Pol θ‐dependent repair outcomes, especially those traditionally classified as having only 1 bp or no MH [8, 34, 38]. Mismatch compatible MH is supported by reanalysis of Pol θ‐dependent repair events in different species, extensive DNA binding contacts in the Pol θ polymerase domain, and extension ability from mismatched primer‐template junctions. Such flexibility is also observed in other (micro)hom(e)ology‐mediated DNA repair pathways such as MMBIR, and NAHR.

However, the pairing information and effective length of MH during TMEJ remain incompletely understood. Although specific primer‐grasp residues of Pol θ polymerase domain and local MH pairing context are known to contribute to MH anchoring, the limited diversity of end‐joining substrates studied so far constrains our understanding. Expanding analyses to a wider range of TMEJ substrates will provide further insights into MH anchoring preferences by Pol θ. During TMEJ, Pol δ removes bases from the 3′ ends of DNA tails, creating new termini that are used by Pol θ to locate a MH and initiate extension [9, 10]. Future work should explore how MH anchoring is modulated by Pol δ or other accessory factors, and how mismatches within repair intermediates are ultimately resolved, as these processes are likely to influence repair outcomes and cell fate decisions.

Pol θ preserves cell viability and maintains genomes at the cost of introducing genomic alterations, including deletions and templated insertions at repair sites. The Catalogue of Somatic Mutations in Cancer (COSMIC) framework provides a resource for interpreting the impact of somatic mutations on cancer development and for presenting available therapeutic options [65]. In HR‐defective circumstances, Pol θ expression level is correlated with the COSMIC small insertion and deletion signature 6 (ID6) and single base substitution signature 3 (SBS3) [46]. ID6 is a deletion‐associated signature characterized by microhomology [66]. As mismatches within MHs are incorporated, the resulting mutational signature landscape of Pol θ is likely to expand beyond current annotated categories. Reanalysis of MH data is also needed in light of the fact that two distinct repair outcomes can arise from a single Pol θ‐mediated MH anchoring.

Breakpoint junctions in the human genome often occur at sites with microhomologies, defined as perfectly matched sequences of 2 – 50 bp [16]. Genome‐wide analysis of virus‐integration showed that around 90% integration products harbor MHs at junctions [67, 68], although the extent to which these events are Pol θ‐dependent remains unclear. Incorporation of mismatches into these MHs would alter the mechanistic interpretation of junction formation and refine how associated mutational signatures are defined. Systematic interrogation of Pol θ‐mediated mutation signatures in cancer genomes may therefore provide further insights into how TMEJ contributes to genomic instability, tumorigenesis, and potential therapeutic vulnerabilities.

## Author Contributions

**Yuzhen Li**: Conceptualization, writing, reviewing, and editing of the manuscript, figures. **Richard D. Wood**: Conceptualization, co‐writing, reviewing, and editing of the manuscript.

## Conflicts of Interest

The authors declare no conflicts of interests.

## Data Availability

Data sharing not applicable to this article as no datasets were generated or analyzed during the current study.
